# PGlu-Modified Nanocrystalline Cellulose Improves Mechanical Properties, Biocompatibility, and Mineralization of Polyester-Based Composites

**DOI:** 10.3390/ma12203435

**Published:** 2019-10-21

**Authors:** Mariia Stepanova, Ilia Averianov, Mikhail Serdobintsev, Iosif Gofman, Natalya Blum, Natalya Semenova, Yuliya Nashchekina, Tatiana Vinogradova, Viktor Korzhikov-Vlakh, Mikko Karttunen, Evgenia Korzhikova-Vlakh

**Affiliations:** 1 Institute of Macromolecular Compounds, Russian Academy of Sciences, St. Petersburg 199004, Russiamkarttu@uwo.ca (M.K.); 2 St. Petersburg Research Institute of Phtysiopulmonology, St. Petersburg 194064, Russia; 3 Interregional Laboratory Center, St. Petersburg 192283, Russia; 4 Institute of Cytology, Russian Academy of Sciences, St. Petersburg 194064, Russia; 5 Institute of Chemistry, Saint-Petersburg State University, St. Petersburg 199034, Russia; 6 Department of Applied Mathematics, The University of Western Ontario, 1151 Richmond str., London, ON N5A 5B7, Canada; 7 The Centre for Advanced Materials and Biomaterials Research, The University of Western Ontario, 1151 Richmond str., London, ON N6A 3K7, Canada; 8 Department of Chemistry, The University of Western Ontario, 1151 Richmond str., London, ON N6A 3K7, Canada

**Keywords:** nanocrystalline cellulose, composite films, polyester materials, mechanical properties, biocompatibility

## Abstract

The development of biocompatible composite materials is in high demand in many fields such as biomedicine, bioengineering, and biotechnology. In this study, two series of poly (D,L-lactide) and poly (ε-caprolactone)-based films filled with neat and modified with poly (glutamic acid) (PGlu) nanocrystalline cellulose (NCC) were prepared. An analysis of scanning electron and atomic force microscopies’ results shows that the modification of NCC with poly (glutamic acid) favored the better distribution of the nanofiller in the polymer matrix. Investigating the ability of the developed materials to attract and retain calcium ions led to the conclusion that composites containing NCC modified with PGlu induced better mineralization from model solutions than composites containing neat NCC. Moreover, compared to unmodified NCC, functionalization with PGlu improved the mechanical properties of composite films. The subcutaneous implantation of these composite materials into the backs of rats and the further histological investigation of neighboring tissues revealed the better biocompatibility of polyester materials filled with NCC–PGlu.

## 1. Introduction

Currently, aliphatic polyesters are one of the most widely used biodegradable polymers. They have found a wide range of applications in fields such as food industry, surgery, drug delivery, and regenerative medicine [1]. One of the main reasons for their popularity is that the synthesis of aliphatic polyesters is simple, reproducible, and inexpensive at the industrial scale. Moreover, their characteristics can be tuned over a wide range. As a result, aliphatic polyesters have become the most commercially competitive polymers. Aliphatic polyesters are biocompatible, biodegradable and approved by the Food and Drug Administration (FDA) agency, and as a result, they are used in diverse applications in medicine [2]. Different materials designs include fibers, microparticles, nanoparticles, films, and porous scaffolds [3,4,5], and poly (lactic acid) and its copolymers with poly (glycolic acid). These are among the most used polyesters in medicine [6,7,8].

Biological inertness and a lack of reactive functionality are among the poor properties of aliphatic polyesters. As a result, more and more research is devoted to their preparation for advanced materials for medical and regenerative applications to overcome the drawbacks arising from potential inflammatory side effects, including their high hydrophobicity, and low cell adhesion [9,10,11]. To eliminate such problems, current approaches include filling polyesters with nanoparticles of different nature [12,13] and their modification with other (macro)molecules of interest [14,15]. 

To reinforce the mechanical properties of polyester materials, different kinds of mineral nanoparticles are typically applied as fillers [12,13,16,17]. However, for the formation of fully renewable, biocompatible, and biomimetic materials, the application of fillers based on nanoparticles and microparticles based on natural polymers is a promising alternative [18,19,20]. In particular, the interest in using nanocrystalline and microcrystalline cellulose (NCC or MCC) as fillers in biodegradable matrices has increased over the last few years [21,22,23]. The low cost and wide availability of cellulose from different sources make it one of the most promising natural materials [24]. However, the direct use of NCC/MCC as filler in aliphatic polyester materials is limited by the interfacial incompatibility of the hydrophilic polysaccharide with the hydrophobic polyester matrix. This obstacle can be overcome by physical or chemical modification of the NCC/MCC surface to achieve better compatibility with aliphatic polyester-based materials [25]. For instance, the adsorption of poly(ethylene glycol) (PEG) on the NCC surface [26] or grafting of poly(lactic acid) (PLA) [27,28] or poly(ε-caprolactone) (PCL) [29] on the NCC surface have been reported. The grafting of PLA/PCL has been carried out via the ring-opening polymerization of L-lactide or ε-caprolactone initiated with the cellulose hydroxyls in the presence of tin (II) octoate. The filling of PLA/PCL films with PLA or PCL-modified NCC/MCC has been shown to improve filler distribution in the polymer matrix and the consequent improvement of mechanical properties of the composites [27,28,29,30].

It has been demonstrated recently that the modification of PLA/poly(lactic acid-co-glycolic acid) (PLGA) nanofibers with glutamic acid peptide induces effective calcium phosphate nucleation and the osteogenic differentiation of marrow stromal cells [31]. The ability to influence mineralization is one of the key features of materials that are candidates to become scaffolds for bone tissue engineering. In this study, we focused on the preparation and characterization of new biocompatible and biodegradable composite materials based on poly (L-lactic acid) (PLLA), poly (D,L-lactic acid) (PDLLA), and PCL filled with NCC particles, which have poly(glutamic acid) (PGlu) grafted onto their surfaces. The mechanical properties, matrix surface morphology, and the ability for mineralization in a model medium, as well as biocompatibility in vitro and in vivo were studied and compared to the results obtained for control materials. 

## 2. Materials and Methods 

### 2.1. Chemicals and Supplements 

All monomers, initiators, and agents for modification were purchased from Sigma-Aldrich (Munich, Germany). NCC is a product of Blue Goose Biorefineries Inc. (Saskatoon, SK, Canada). Organic solvents (toluene, chloroform, methanol, etc.) that were used for polymer synthesis, modification, and the preparation of films were from Vecton (St. Petersburg, Russia). All solvents were distilled before application. The THF used as eluent for size exclusion chromatography (SEC) analysis was of HPLC grade and purchased from Merck (Darmstadt, Germany). PGlu was synthesized in IMC RAS via the ring-opening polymerization of γ-O-benzyl-α-glutamic acid N-carboxyanhydride, and then deprotected with TFA/TFSA, as described elsewhere [32,33]. The characteristics of PGlu were the following: *M_w_* = 2100 (weight average molecular weight), *M_n_* = 2000 (number average molecular weight), *Ɖ* = 1.05 (dispersity), and the amount of residual benzyl groups was 22% (according to NMR) [34]. Polystyrene (PS) calibration standards with molecular weights from 2000 to 400,000 were purchased from Waters (Milford, MA, USA). Before chromatographic analysis, sample solutions were filtered through a Millipore Merck syringe PTFE membrane filter with a 0.45-μm pore diameter (Darmstadt, Germany).

### 2.2. Methods

#### 2.2.1. Synthesis and Characterization of Polymers

The synthesis of PDLLA and PCL was carried out in bulk at 130 °C via the ring-opening polymerization of D,L-lactide and ε-caprolactone, respectively. The composition of polymerization mixture and polymerization time was optimized to obtain polymer samples with close intrinsic viscosity. The monomer-to-Sn (Oct)_2_ ratio was equal to 2500 for PDLLA and 5000 for PCL. In the case of PCL synthesis, methanol was added to the polymerization mixture ([Methanol]/[Sn(Oct)_2_] = 2). Synthesis was conducted in vacuum-processed Schlenk tubes over 7 h for PDLLA and 20 h for PCL. After synthesis, polymers were dissolved in chloroform, precipitated in cold methanol, filtered and washed with a fresh portion of methanol, and dried in vacuum. The molecular weights (*M_w_*) and dispersity (*Ɖ*) were determined by size exclusion chromatography (SEC) with the use of a Shimadzu HPLC system (Shimadzu Corporation, Tokyo, Japan) consisting of a pump LC-10AD VP, system controller SCL-10A VP, and refractometric detector RID-10A (Canby, OR, USA) supplied with a Rheodyne 725i injection valve (Rheodyne, Rohnert Park, CA, USA) and two columns of Agilent PLgel MIXED-D (7.5 × 300 mm, 5 µм) (Santa-Clara, CA, USA). Analyses were performed at 40 °C and at a flow rate of 1.0 mL/min with the use of THF as an eluent and PS standards for calibration. Data acquisition and processing was performed using LC solution software (version 1.25, Shimadzu Corporation, Kyoto, Japan). Viscosities of sample solutions (in CHCl_3_) with different concentrations were measured using Ostwald’s capillary viscosimeter. 

#### 2.2.2. NCC Modification with PGlu 

Before modification, the NCC suspension (8 mass%) was diluted 10 times with water and cooled to 4 °C. Then, the aqueous solution containing 300 mg of NaIO_4_ was added to the suspension containing 1 g of NCC. The reaction was carried out at 4 °С for 24 h. The oxidized NCC was purified by dialysis against water with the use of the membrane bag with MWCO (molecular weight cut off.) 1000. The product was stored in suspension at 4 °C. Prior to use, the product was concentrated on a rotary evaporator. The concentrated suspension was added to an excessive amount of buffer solution of choice, so that the pH was not changed in the tenths place value. 

Then, 250 mg of PGlu in 10 mL of water was added to 100 mL of suspension containing 1.0 g of oxidized NCC in 0.01 M sodium borate buffer (pH 8.5). The reaction was carried out for 3 h at 22 °C under stirring. To reduce imine bonds and free aldehyde groups, 200 mg of sodium borohydride was added to reaction medium that was left for 1 h. 

#### 2.2.3. Manufacturing of Films

Cellophane, moistened with water, was fixed on the glass cylinder (i.d. = 75 mm) and then dried for 24 h. A 5% polymer solution in chloroform (6.5 mL) was poured onto cellophane inside the glass cylinder and left for 12 h in air for chloroform evaporation. After that, the cellophane was removed, and the obtained polymer films were dried in air thermostat at 50 °C until constant weight was reached (3 days for PCL, and 7 days for PDLLA). 

To prepare composite films, original or modified NCC was dispersed in polymer solution in chloroform under ultrasonication. The amount of NCC was equal to 5%, 10%, and 15% regarding the weight of polymer in solution. Other manipulations were done as described above for preparations of pure polymer films.

#### 2.2.4. Study of Mechanical Properties

Mechanical characteristics of the films were determined under uniaxial extension using band-like specimens of 2 mm × 20 mm using the AG-100kNX Plus Shimadzu universal mechanical system (Tokyo, Japan). The extension speed was 10 mm/min. 

#### 2.2.5. Microscopy and X-Ray Phase Analysis

Surface morphologies and topographies were analyzed by scanning electron (SEM) and atomic force microscopy (AFM) using Zeiss AURIGA Laser (Oberkochen, Germany) and INTEGRA-Aura Spectrum Instruments (Moscow, Russia), respectively. Before the registration of SEM images, the surfaces of film specimens were additionally deposited with conductive carbon layers. The AFM images were analyzed with the WSxM software (version 4.0, Julio Gómez Herrero & José María Gómez Rodríguez, Madrid, Spain) [35]. Polarized light microscopy was carried out by means of a Leica DM4500 P instrument (Leica Microsystems, Wetzlar, Germany) in transmittance mode. The Rigaku MiniFlex II system (Tokyo, Japan) was applied for the analysis of crystallinity of pure and composite film specimens.

#### 2.2.6. Cell Culture Experiments 

Cell adhesion on the surfaces of the obtained films was evaluated using bone marrow mesenchymal stem cells (MSC). Mesenchymal bone marrow stromal cells were isolated from newborn rabbit bones. The rabbits were purchased from the Federal State Unitary Company “Nursery Rappolovo”. The procedure of obtaining MSC from rabbits was approved in an Institute of Phthysiopulmonology Ethical Committee session and protocoled (Statement #46 from 18.04.2018). MSCs were cultivated in αMEM medium (Lonza, Allendale, NJ, USA) supplemented with 10% fetal bovine serum (FBS) (HyClone, GE Healthcare Life Sciences, Chicago, IL, USA), 100 U/mL penicillin (Sigma-Aldrich, WGK Germany), and 100 μg/mL streptomycin (Sigma-Aldrich, Darmstadt, Germany). In experiments, the cells of 2–6 passages were used. Cell cultivation was performed in a thermostatic incubator (5% CO_2_, 37 °C). The round-shaped films with a diameter of 6 mm were stuck into 96-well plates and MSC (5 × 10^3^ cells per well) were seeded. After 24 hours of incubation, the non-attached dead cells were washed out, and the amount of attached cells was evaluated using an MTT test. The data from cell culture experiments are presented as average ± SD (*n* = 4). 

#### 2.2.7. Mineralization Study 

The experiments were carried out using simulated media containing Ca^2+^ and PO_4_^3−^ ions at concentrations known for body fluids [36]. For each polymer, a set of materials containing pure and composite film specimens manufactured with original and modified NCC was utilized for the study. 

Before the experiments, round-shaped film specimens of 5 mm in diameter and about 100 μm in thickness were incubated in 50% ethanol solution in water (*v*/*v*) at room temperature for 1 h to achieve better material surface wettability. Then, the specimens were washed with water, placed into separate wells of a microtiter plate, and 1 mL of 3 mM aqueous CaCl_2_ solution was added into each well containing the testing material. The microtiter plate was incubated for 5 days, and then the medium was replaced with water to wash the specimens. Washing was carried out for 2 days. After that, water was replaced with 3 mM of aqueous NaH_2_PO_4_ solution, the specimens were incubated for 5 days, and then again, they were washed with water for 2 days. The temperature of the process at all steps was 37 °C. The described cycle took 2 weeks and was repeated 15 times. After final careful washing, the tested materials were dyed with alizarin red S. For this, the specimens were inserted into 2% aqueous dye solution for 40 min and washed up to colorless washing solutions. The images were registered by an optical microscope Nikon Eclipse E200 (Tokyo, Japan).

#### 2.2.8. In Vivo Experiments 

All experimental procedures with animals were organized and maintained fulfilling the principles of humane care of animals with the consent and approval of the Bioethical Review Committee of the Research Institute of Pthisiopulmonology (Statement #46 from 18.04.2018). Twenty-one male rats (Wistar line, 11 weeks old, average weight 311.1 ± 15.7 g) were purchased from the Federal State Unitary Company “Nursery Rappolovo” and used in the experiments. Animals prior to the study were quarantined for 14 days and monitored daily by visual clinical inspection. Only clinically healthy rats were included in the experiments. They were given standard diet fortified in protein and vitamins (Furage Ltd., St. Petersburg, Russia), as recommended by Russian Health Ministry edict #1179 for animal experimentation, plus water ad libitum. The rats were kept at the vivarium of Research Institute of Phthisiopulmonology under standard conditions in full accordance with the “Rules of an establishment, equipment and maintenance of experimental biological clinics”, approved by Russian Federation National standard R53434-2009 and by the “European Convention for the Protection of Vertebrate Animals used for Experimental and other Scientific Purposes” (CETS No. 123, 1991). The light conditions were 12 h light/12 h darkness, air temperature was maintained between 23–25 °C, and relative air humidity was 50–70%. Air exchange (12–15 volumes/hour) was carried out by inflow–outflow ventilation, with daily air sterilization by UV light. The rats were housed in groups of three animals in polycarbonate cages, with a floor area of 600.0 cm^2^ per animal.

Scaffold preparation. The round-shaped film specimens with 5 mm in diameter and about 100 μm in thickness were prepared from larger films (see Section 2.2.3) with the application of mandrel and stored at 4 °C. Just before implantation, films were sterilized by incubation in 70% ethanol solution for 1 hour and then repeatedly washed 10 times with PBS. The sterilization was performed in a laminar flow box “Laminar-C”-1,2-ME (EuroLab, St. Petersburg, Russia).

**Figure 1:**
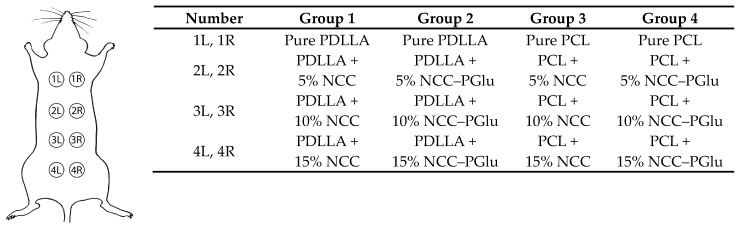
Scheme of implantation of the specimens and groups of animals. Each group included three rats to whom the specimens were implanted twice, e.g. on the left and the right side from the backbone of the same animal. The total number of repeats for composite materials was 6; for pure aliphatic polyester specimens, it was 12. NCC: nanocrystalline cellulose, PCL: poly(ε-caprolactone), PDLLA: poly (D,L-lactic acid), PGlu: poly(glutamic acid).

Scaffold implantation. The rats were randomly divided in 6 experimental groups and 1 control group with 3 animals per group (n = 3, see Figure 1). The tested samples were implanted into the subcutaneous tissue of the rat’s back along the spine on the right and left, following the rules of aseptic operation. Eight samples were implanted into each animal (4 on each side of the back, of which 1 control and 3 experimental). All manipulations were performed under general anesthesia using a 0.2 wt.% solution of rometar (Xylazinum, Biovet, Czech Republic) at a dose of 0.1 mL per 100 g of animal weight. The anesthetic was injected into the thigh muscle of the rats. For the implantation procedure, the rats were fixed on a surgical operation table DG-s 3W Fengshi (Open Science, Russia) in the position “on abdomen”. The hair on the back was carefully sheared, and this area was treated with 5% alcohol solution of iodine. To implant the scaffolds, 10-mm incisions were cut on the dorsal section of rats. Then, the incisions were sutured using a poly (glycolic acid) (PGA) retention suture (Lintex, Russia) and Cefamesin^®^ (20 mg/kg, intramuscularly, dissolved in solution of 0.25 wt.% Procaine, 5 days; Pharm-Sintez, Russia) was applied to prevent postoperative complications. The rats were monitored daily during the whole experiment, which ran for 4 weeks. The weight of the animals was used as a parameter to control the health of the animals.

Scaffold resection. At the end of the experiment, the animals were euthanized with Zoletil^®^ (Virbac, Carros, France) (total dose—60 mg/kg; active components: tyletamine hydrochloride and zolazepam hydrochloride; Virbach CA, France). A macroscopic study of model animals was carried out via an inspection of the implantation sites. The skin was sheared around the marked implantation site; then, skin pieces were cut and fixed in 10% formalin solution for at least 48 hours. Then, the samples were kept in 70% ethanol before being embedded in paraffin by the Interregional Laboratory Center (St. Petersburg, Russia).

#### 2.2.9. Histomorphometric Analysis

Skin sections at the implantation places were cut together with neighboring tissues. All specimens were fixed with 10% formalin buffer solution (рН 7.4) for 24 h and embedded in paraffin using an automatic Excelsior AS Tissue Processor, ThermoFischer Scientific (Waltham, MA, USA) and commercial medium IsoPREP Biovitrum (St. Petersburg, Russia). Finally, the specimens were impregnated with HISTOMIX medium, Biovitrum (St. Petersburg, Russia), and the obtained blocks were sliced into 3-μm thick sections by rotary microtome НМ 325 ThermoFisher Scientific (Waltham, MA, USA). The prepared slices were stained with hematoxylin-eosin according to the protocol of the manufacturer Biovitrum (St. Petersburg, Russia) and analyzed with the use of transmitted-light bright field AxioLab Zeiss microscope (Carl Zeiss, Jena, Germany). 

Morphometric study was carried out using a Carl Zeiss Axio Imager AZ microscope (Jena, Germany) supplied with the Axio Vision software (version 4.8, Carl Zeiss, Jena, Germany) for the analysis of images. To verify the histological changes, histoarchitectonics of adjacent tissues were evaluated, and the thicknesses of the capsules formed around the implants were also measured. The morphometric characteristics in the studied area were determined for the following cell populations: fibroblasts, lymphocytes, neutrophils, eosinophils, and giant cells. Additionally, the number of vessels and layers of collagen fibers in the capsule were also calculated.

The analysis was carried out in 10 fields of view in areas oriented in the same plane. The average parameter value for each sample and then the average parameter value for the group were calculated. The data were analyzed using Student’s test (t-test), and p < 0.05 was set as the level of statistical significance.

## 3. Results and Discussion

### 3.1. Synthesis of Matrix Polymers and Manufacturing of Composite Fillms 

PDLLA and PCL were synthesized by the ring-opening polymerization of D,L-lactide and ε-caprolactone, respectively, in presence of tin (II) octoate. The polymerization conditions were chosen to obtain the polymers suitable for the further preparation of casting films. In order to obtain comparable films based on PDLLA and PCL, the values of their solutions’ intrinsic viscosities should be close. This provides comparable solution casting and allows unifying the films’ thickness. 

The yields of polymer products and their characteristics are summarized in Table 1. The molecular weight and dispersity of polymers were determined via size-exclusion chromatography in THF at 40 °C regarding the polystyrene standards. The characteristic viscosity was determined using the Ostwald’s viscosimeter. As seen from the data presented in Table 1, the values of intrinsic viscosities were satisfactory for the further manufacturing of films via the solution-casting approach. In addition, these values are close to each other.

**Figure 2:**
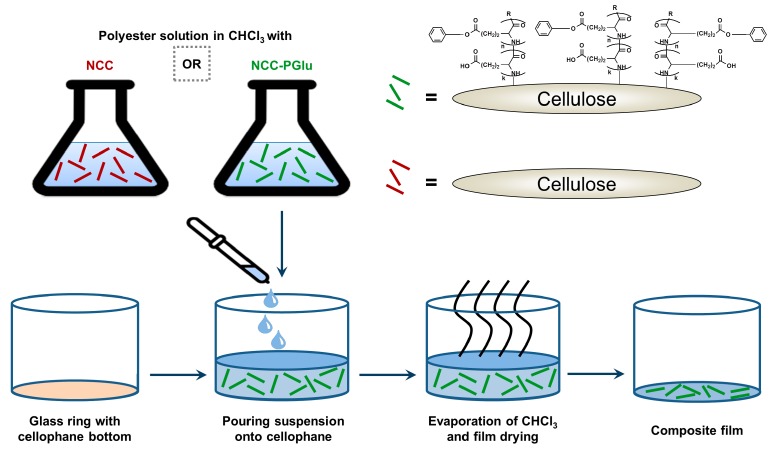
General scheme for the preparation of composite films.

The manufacturing of films was carried out via casting of the polymer solution in chloroform onto the cellophane support and further solvent evaporation. The general scheme of composite film preparation is illustrated in Figure 2. After the evaporation of chloroform at room temperature, the films were additionally dried at 50 °C to remove solvent traces. The films were dried until constant weight. It took 3 days for PCL and 7 days for PDLLA. 

According to transmission electron microscopy, the cellulose nanocrystals used in this work were 150 nm in length and 10 nm in diameter (information provided by manufacturer). Also, NCC was characterized by negative electrokinetic potential, which was measured to be –23 mV. The evaluation of commercial NCC suspension by dynamic light scattering allowed the detection of the NCC hydrodynamic diameter as around 130 nm. The lyophilization of commercial NCC suspension and further redispersion in aqueous media was followed with the increase in hydrodynamic diameter of NCC up to 265 ± 20 nm. 

**Scheme 1:**
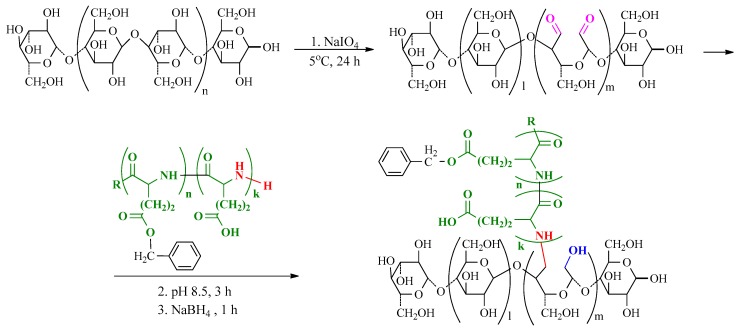
The modification of NCC with PGlu consisted of three steps as indicted: (1) NCC oxidation to generate highly reactive aldehydes; (2) Reaction between aldehydes and terminal amino group of PGlu with the formation of imine (Schiff’s base) bonds; (3) Imine bonds and unreacted aldehyde groups were reduced.

The pure polymer films were prepared as benchmarks for monitoring the effect of NCC addition. Neat NCC and NCC modified with PGlu were used as fillers at concentrations of 5, 10 and 15 mass%. To reach a better compatibility of hydrophilic NCC–PGlu with the polyester matrix, the deprotection of PGlu(OBzl) after synthesis was carried out incompletely to retain part of the hydrophobic benzyl units. The amount of residual benzyl moieties was 22 mol%. The procedure of NCC modification with PGlu has been recently developed in our group (Scheme 1) [34]. 

The optimal modification route includes three steps. First, NCC oxidation is carried out to generate highly reactive aldehydes. Second, the reaction between aldehydes and the terminal amino group of PGlu with the formation of imine (Schiff’s base) bonds is performed. Finally, the imine bonds and the unreacted aldehyde groups are reduced with sodium borohydride. The modification efficiency was evaluated [34] to be 225 ± 13 mg/g of NCC, which corresponded to 90 ± 6% of the amount of initial PGlu taken for the reaction. Both NCC oxidation and the modification of NCC with PGlu were accompanied by an increase in NCC hydrodynamic diameter of up to 360–390 nm. Additional information on NCC modification as well as other characteristics of neat NCC and NCC–PGlu can be found in our earlier work [34]. 

As expected, the dispersion of the neat NCC in the polymer solution prepared in chloroform was followed by an aggregation of hydrophilic filler. Extensive suspension ultrasonication before film casting was required in order to obtain a more or less homogeneous dispersion of crystals in the polyester matrix. In spite of this, aggregates were still present in the final films due to the poor compatibility of the hydrophobic matrix polymer and hydrophilic NCC. In the case of NCC modified with PGlu, aggregation was much less pronounced, and NCC distribution in the polymer matrix was much more even. 

### 3.2. Physicochemical Characterization of Composite Materials 

#### 3.2.1. Surface Morphology and Topography

**Figure 3:**
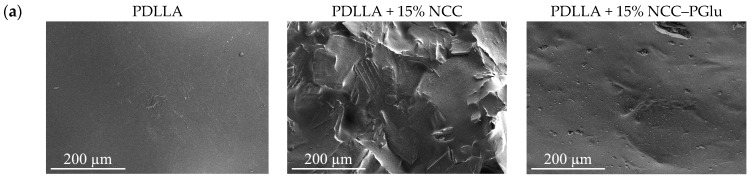

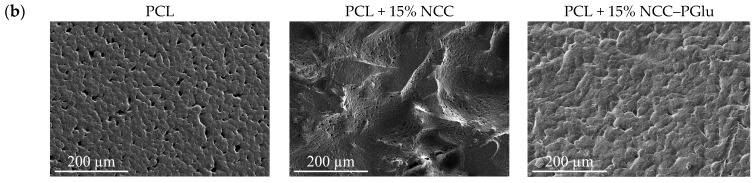
SEM images of PDLLA-based (**a**) and PCL-based (**b**) pure and composite films (500×).

Figure 3 illustrates the SEM images registered for the surfaces of pure and composite films based on PDLLA and PCL. They are the amorphous and crystalline polymers, respectively. The morphologies of the manufactured pure PDLLA and PCL depended on a polymer type: Films based on PDLLA possess enough smooth dense surface, while the PCL-based films, characterized by high relief surface, are pierced with a certain amount of pores. The formation of porous materials for crystalline polymers has been reported before [37,38]. The morphological features of the samples based on pure polyester and composite films are in good agreement with the data obtained by X-ray diffraction analysis (XRD) (see Supplementary Materials). 

**Figure 4:**
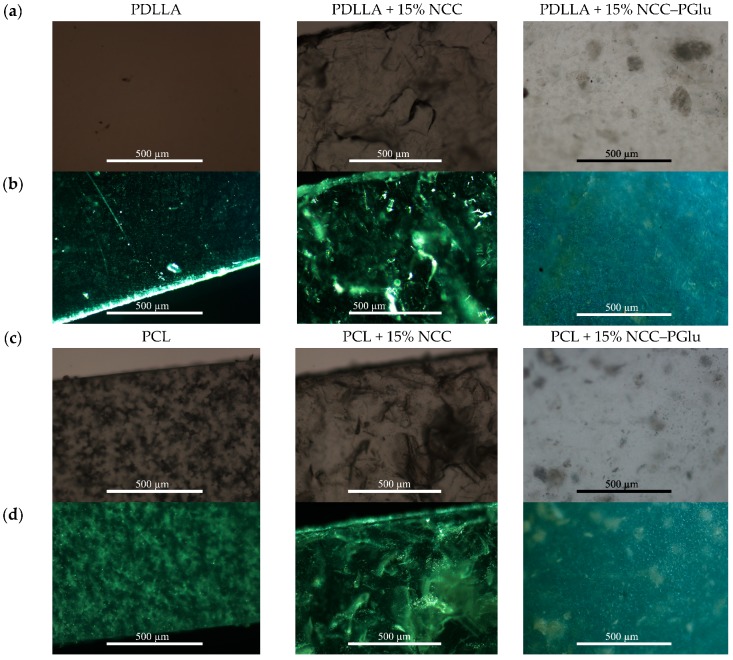
Optical transmitted light (**a**,**c**) and reflected (**b**,**d**) microscopy images registered for pure PDLLA and PCL and their composites with neat and modified NCC (10×).

The formation of pores is provoked by crystallization of the polymer during the volatile organic solvent evaporation. The comparison of images registered for films manufactured from pure polymer and polymer containing 15 mass% of non-modified NCC (Figure 3 and Figure 4) shows considerable surface roughness in the second case. 

The casting of composite films from polymer solution containing the same amount of modified NCC provided much better surface uniformity comparable with polymer films without filler. Additionally, the obtained pure polyester and composite materials were investigated by optical transmitted light and reflected microscopy (Figure 4). For both PDLLA and PCL, the observed surface morphology was in agreement with SEM images: The materials containing neat NCC were characterized by higher roughness provided by the larger size of the aggregates. 

**Figure 5:**
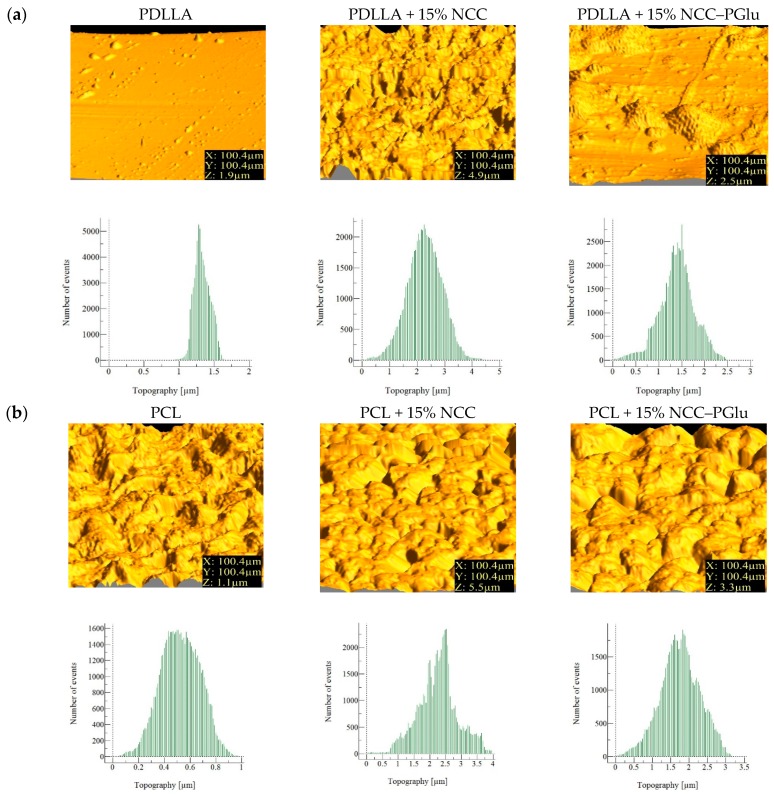
Atomic force microscopy (AFM) images and roughness profile of the images for PDLLA-based (**a**) and PCL-based (**b**) pure and composite films.

Surface topography of the prepared materials was evaluated by AFM (Figure 5). For the PDLLA, PDLLA + 15% NCC, and PDLLA + 15% NCC–PGlu specimens, the average heights were 1.3 μm, 2.3 μm, and 1.5 μm, respectively. In turn, the average heights for PCL, PCL + 15% NCC, and PCL + 15% NCC–PGlu specimens were found to be 0.5 μm, 2.5 μm, and 1.8 μm, respectively. Additionally, the root mean square (RMS) roughness was calculated. The RMS values for PDLLA, PDLLA + 15% NCC, and PDLLA + 15% NCC–PGlu specimens were equal to 0.12 μm, 0.78 μm, and 0.44 μm, respectively. At the same time, for the PCL, PCL + 15% NCC, and PCL + 15% NCC–PGlu materials, the RMS roughness values were calculated as 0.15 μm, 0.75 μm, and 0.55 μm, respectively. From the results obtained, it is obvious that the roughness of the composite materials is higher than that of the pure polymer films. However, the average heights and RMS values calculated for composite materials containing modified NCC were lower than those for neat NCC, demonstrating the better compatibility of modified NCC with polyester matrices.

#### 3.2.2. Mechanical Properties

Mechanical characteristics of the films were determined under uniaxial extension using band-like specimens of 2 mm × 20 mm. Young’s moduli *(E)*, tensile strengths (*σ_b_*), and elongations at break (*ε_b_*) were determined for pure PDLLA and PCL films as well as their composites with NCC and NCC–PGlu (Table 2). 

The addition of unmodified NCC to PDLLA was accompanied by a decrease in Young’s modulus by 30%, whereas *E* values were not changed for PCL-based films filled with neat NCC. For elongation at break, a drastic reduction for both PDLLA and PCL composites was observed. Obviously, the introduction of filler led to the formation of zones with accentuated fragility. This is due to the material inhomogeneity caused by the formation of NCC aggregates.

In turn, the application of NCC modified with PGlu as filler to both PDLLA and PCL films was followed by an increase in Young’s modulus values due to the better nanofiller–matrix interfacial interactions. The tensile strength remained unchanged for all PDLLA-based composites. At the same time, for PCL-based composites, this parameter decreased more than 3 times for neat NCC and only twice for NCC–PGlu. 

The obtained results allow us to conclude that the application of unmodified NCC leads to a drastic change in the properties due to a lack of compatibility between the unmodified cellulose nanocrystals and the matrix. A similar tendency was previously observed when we compared pure PCL films with PCL-based films filled with neat NCC and NCC grafted with PLA [30]. The modification of NCC with PGlu improves the mechanical properties of films in comparison with the composites containing neat NCC. Despite the decrease in tensile strength and elongation at break values in comparison with pure polymer films, PDLLA and PCL composites with NCC–PGlu have still acceptable mechanical properties. At the same time, the composites with modified NCC surpass pure polymer films in other properties such as mineralization and biological properties (see below). 

### 3.3. In Vitro Mineralization 

**Figure 6:**
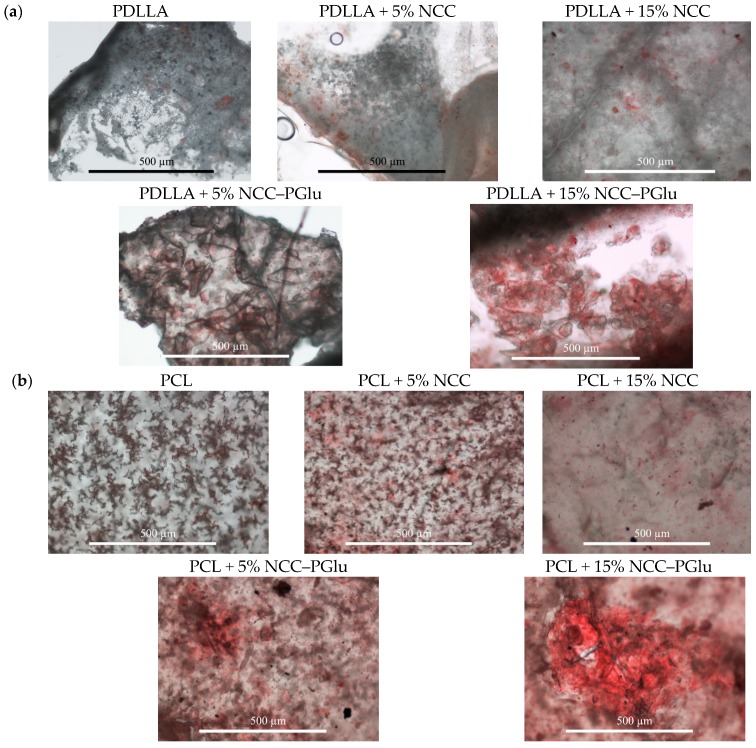
In vitro mineralization study of the pure PDLLA (**a**) and PCL (**b**) materials and their composites with original and modified NCC. The mineralization study was performed by alizarin red S assay. The intensity of the red color corresponds to the amount of calcium ions on the surface of the films.

The investigation of the abilities of the composite materials to mineralize was carried out with the use of two model solutions containing Ca^2+^ and PO_4_^3−^, respectively. The sequential exposure of the film specimens in Ca^2+^-containing or in PO_4_^3−^-containing solutions was performed for 30 weeks. The evaluation of mineralization was performed by alizarin red S staining. As seen from Figure 6, pure aliphatic polyester materials did not show staining with alizarin red S. This indicates the absence of calcium deposits. For composites, less mineralization was observed in the case of PDLLA and PCL materials filled with neat NCC, whereas remarkably higher mineralization was noted in the polymer materials containing NCC–PGlu. 

Thus, the modification of NCC with PGlu favored not only better mechanical properties but also the improvement of the Ca^2+^ binding by the composite material. The biocompatible and anionic nature of NCC–PGlu provided the formation of well-distributed mineral deposits in the scaffold material. Therefore, the developed NCC–PGlu-containing composites, which could induce mineralization, are interesting as prospective materials for bone tissue engineering applications.

### 3.4. Biological Evaluation 

#### 3.4.1. In Vitro Study

**Figure 7:**
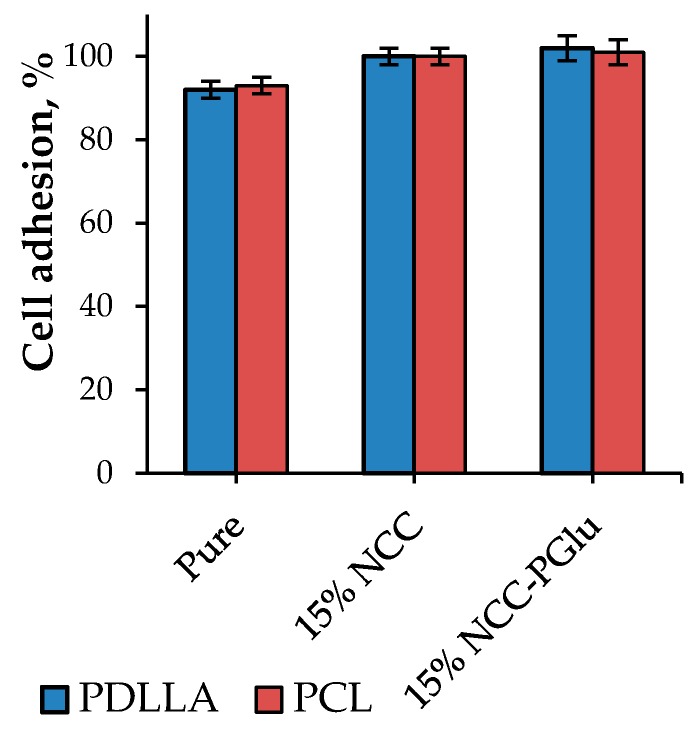
MTT test: Comparison of mesenchymal stem cells (MSC) cell adhesion on the surface of different materials. (Conditions: 24 h of cultivation, 5 × 10^3^ cells per well. Data presented as average ± SD (n = 4))

First, we studied the compatibility of obtained materials with cells. Figure 7 illustrates the dependence of MSC cell adhesion on material composition. In all cases, the measured parameters were very close to each other, indicating comparable cell adhesion onto both original polyester materials and composite films. Thus, the introduction of NCC into PCL as well as the discussed modification of NCC did not make materials toxic for the cells. Moreover, the obtained results prove that polyester materials filled with NCC–PGlu offer excellent biocompatibility for bone tissue regeneration.

#### 3.4.2. Biocompatibility in Vivo

A set of round-shaped specimens with a diameter of 5 mm and 100 μm in thickness were implanted into the subcutaneous pockets on the back of each rat to test the biocompatibility of the composite materials in vivo. The implantation scheme and groups of animals are presented in Figure 1. The specimens were implanted under the muscular layer of the dermis. After four weeks, the scaffolds were resected from the animals, and histological and morphometric analysis was carried out. 

**Figure 8:**
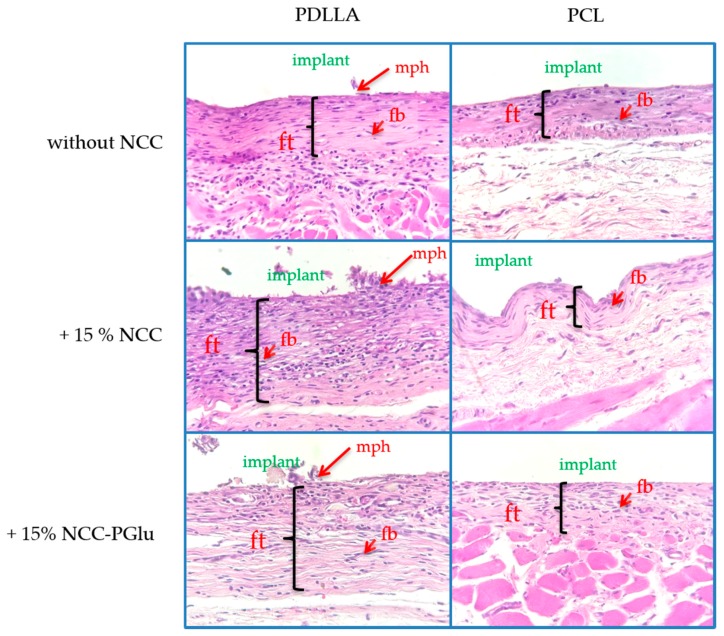
Representative histological sections (×40) after four weeks of subcutaneous implantation. (Abbreviations: mph – macrophage, fb – fibroblast, ft – fibrous tissue.).

Typical histological sections of materials after subcutaneous implantation are shown in Figure 8. All films led to the formation of foreign body encapsulation during the implantation period of 4 weeks. In all images, one can observe immune cells, macrophages, and supergiant multinuclear cells, as well as fibroblasts. Collagen fibers are also well visualized within the sections. Overall, the histological images show a normal reaction toward implanted material. It is notable that all implanted materials exhibited similar effects on surrounding tissues, involving the thickness of surrounding fibrous capsules, immune cells, and fibroblasts contents. Thus, histological observations clearly demonstrate that none of the tested materials provoked acute toxicity. 

**Figure 9:**
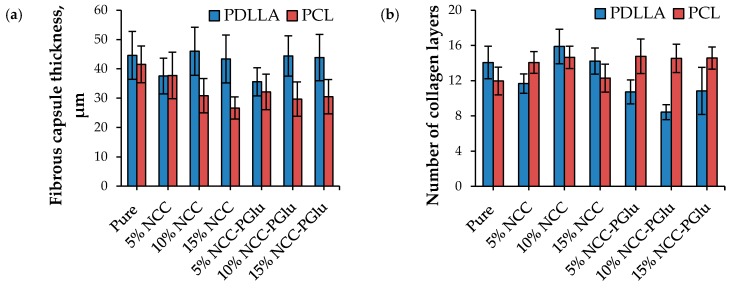

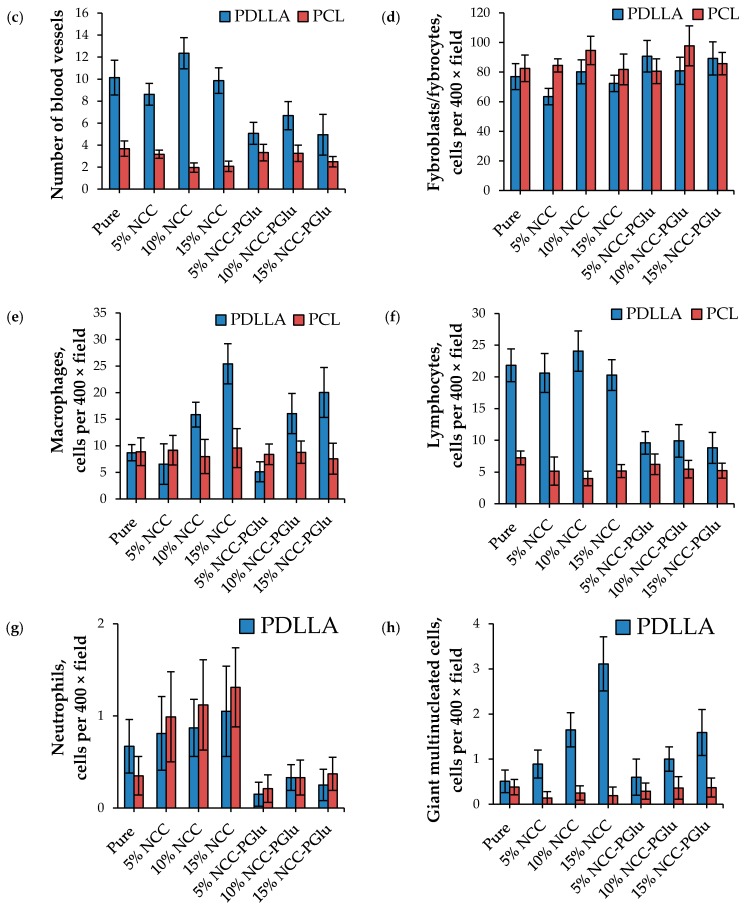
Results of histological analysis after subcutaneous rat implantation with pure aliphatic polyester-based film specimens and these filled with neat and modified NCC: (**a**) the thickness of fibrous capsule, (**b**) and (**c**) the average number of collagen layers and blood capillaries, (**d**–**h**) the average number of different cells per 400× field, e.g. fibroblasts, macrophages, lymphocytes, neutrophiles, and giant multinucleated cells.

Furthermore, the quantitative results of histological examination were compared. The results for different biological components located within the tissues close to the surface of implanted material are presented in Figure 9. These data allowed the comparison of regeneration features after implantation.

Fibrous capsule formation reflects the final step of healing of a wound generated by implantation, and the capsule thickness indicates the biocompatibility of the material in the body. It is postulated that a material with better biocompatibility produces a thinner capsule [39,40]. In our case, microscopic observation revealed that thin capsules with thicknesses varying from 27 to 46 μm had formed around all the implanted specimens for both PLA and PCL-based materials (Figure 9a). However, the fibrous capsules formed around the PLA-based materials (35–46 µm) were slightly thicker than those observed around the PCL-based implants (27–41 µm). At the same time, no evident correlation between the NCC content in the aliphatic polyester matrixes and fibrous capsule thicknesses was detected (p < 0.1). Collagen fibrillogenesis was comparable for all PCL and PDLLA-based specimens, and the average number of collagen fibrils was in the range from 9 to 16. In turn, the number of blood vessels was higher for the pure PDLLA and PDLLA + NCC composites than for the PCL-based ones. Among the PDLLA-based matrixes, the PDLLA + NCC demonstrated higher number of vessels in comparison with the corresponding composition of PDLLA + NCC–PGlu materials (p < 0.05).

A histomorphometric analysis of resected specimens allowed the detection of different cells, e.g., fibroblasts, macrophages, lymphocytes, neutrophils, and giant multinucleated cells. Fibroblasts are responsible for the production of components of the extracellular matrix and the regeneration process. For all analyzed specimens, the average number of fibroblasts was found to be comparable, indicating similar wound-healing rates. The average number of macrophages was equal for all PCL-based materials and close to pure PDLLA films and those filled with 5% NCC or NCC–PGlu. The increase of filler concentration up to 10–15% in the PDLLA-based composite led to a minor rise in the number of macrophages in the implantation area (p < 0.05). 

All PCL-based implants demonstrated low values very close to the average number of lymphocytes. The same was observed for PDLLA films filled with NCC–PGlu. In turn, pure PDLLA and PDLLA filled with unmodified NCC demonstrated the increased number of lymphocytes (p < 0.01). As to neutrophils, the lowest average numbers were detected for PCL and PDLLA composites filled with modified NCC (p < 0.05). Some amounts of giant multinucleated cells were also detected in all specimens. However, their level in pure PDLLA and all PCL-based materials was much lower than in PDLLA-based composites. The highest number of giant multinucleated cells was detected in PDLLA filled with 10% and 15% NCC (p < 0.01). However, there were no statistical differences between the PDLLA and PDLLA–NCC–PGlu (p = 0.2).

In general, it can be concluded that pure PCL as well as its composites demonstrated lower inflammatory response than PDLLA-based materials. The higher inflammation effect detected for PDDLA-based materials can be a result of their faster degradation and stronger acidification of the environment in comparison with PCL matrices. A comparison of the average numbers of lymphocytes, neutrophils, and giant multinucleated cells for PDLLA composites allows the conclusion that films containing neat NCC induced a higher inflammation response regarding PDLLA filled with NCC–PGlu. 

## 4. Conclusions

We have developed polyester-based materials filled with NCC–PGlu, and demonstrated their improved mechanical characteristics compared to neat NCC as a filler. These data are explained by the better distribution of modified NCC in the hydrophobic aliphatic polyester matrix and interactions between modified crystals and the polymer matrix. This explanation is also supported by the surface morphology and topography results. Moreover, the manufactured composites of PE and NCC–PGlu demonstrated better uptake and retention of Ca^2+^ ions. The in vitro and in vivo studies indicate the high biocompatibility and suitability of the composites for biomedical applications. It is important to note that none of the composite materials caused any necrotic processes in the surrounding tissues, and did not demonstrate an elevated expression of inflammatory cells in comparison with pure aliphatic polyester. Moreover, the composites based on aliphatic polyester and NCC–PGlu showed lower inflammatory response than pure aliphatic polyester or composites filled with neat NCC. 

## Figures and Tables

**Table 1:** Characteristics of the matrix polymers used for the preparation of composite materials.

Polymer	Polymer Yield, %	Polymer Characteristics		
		*M_w_*	*Ɖ*	*η (CHCl_3_)*, dL/g
PDLLA	67	162,300	1.37	1.1
PCL	81	155,000	1.53	1.3

**Table 2:** Mechanical properties of pure and NCC and NCC–PGlu filled films (based on PDLLA and PCL).

Film	*E*, GPa	*σ_b_*, MPa	*ε_b_*, %
PDLLA	1.62 ± 0.06	35 ± 2	67.0 ± 7.0
PDLLA + 5 wt% NCC	1.12 ± 0.08	32 ± 2	4.9 ± 0.3
PDLLA + 10 wt% NCC	1.25 ± 0.06	30 ± 1	2.9 ± 0.1
PDLLA + 15 wt% NCC	1.16 ± 0.03	21 ± 2	2.2 ± 0.2
PDLLA + 5 wt% NCC–PGlu	2.82 ± 0.30	31 ± 1	4.8 ± 0.6
PDLLA + 10 wt% NCC–PGlu	2.66 ± 0.29	32 ± 1	3.4 ± 0.2
PDLLA + 15 wt% NCC–PGlu	2.44 ± 0.21	31 ± 1	3.7 ± 0.2
PCL	0.33 ± 0.01	25 ± 3	834.0 ± 65.0
PCL + 5 wt% NCC	0.35 ± 0.03	8 ± 1	19.2 ± 5.1
PCL + 10 wt% NCC	0.34 ± 0.02	7 ± 1	8.8 ± 1.3
PCL + 15 wt% NCC	0.32 ± 0.01	5 ± 1	9.1 ± 1.0
PCL + 5 wt% NCC–PGlu	0.51 ± 0.01	13 ± 1	25.0 ± 5.0
PCL + 10 wt% NCC–PGlu	0.57 ± 0.03	14 ± 1	17.3 ± 3.2
PCL + 15 wt% NCC–PGlu	0.58 ± 0.01	11 ± 1	8.6 ± 0.5

## Supplementary Materials

The following are available online at https://www.mdpi.com/1996-1944/12/20/3435/s1, Figure S1: XRD patterns of pure PDLLA and PCL, and their composites with original and modified NCC.
