# Supplementary material for: PGlu-Modified Nanocrystalline Cellulose Improves Mechanical Properties, Biocompatibility, and Mineralization of Polyester-Based Composites
Source: Materials (Basel). 2019 Oct 21;12(20):3435. doi: 10.3390/ma12203435 (PMC6829302; doi:10.3390/ma12203435)
Supplement: Supplementary file 1 [file materials-12-03435-s001.pdf]

materials

Supplementary

PGlu-Modified Nanocrystalline Cellulose Improves Mechanical Properties, Biocompatibility, and Mineralization of Polyester-Based Composites

Mariia Stepanova [1], Ilia Averianov [1], Mikhail Serdobintsev [2], Iosif Gofman [1], Natalya Blum [3], Natalya Semenova [3], Yuliya Nashchekina [4], Tatiana Vinogradova [2], Viktor Korzhikov-Vlakh [1,5], Mikko Karttunen [1,6,7,8] and Evgenia Korzhikova-Vlakh [1,5,*]

Crystallinity Evaluation

As expected, no diffraction reflexes were observed for PDLLA, indicating the amorphous nature of the polymer. The broad halo at 2θ equal to 10.0–25.0° was detected. The addition of NCC led to the appearance of the characteristic peak near 23.8° against the background of an amorphous halo. In turn, PCL specimens were characterized with the presence of fairly pronounced diffraction peaks located at 21.4°, 22.1°, and 23.8°. The results are coincided with previously published data [1,2]. This indicates the semi-crystalline nature of the polymer and composite films. The contents of the crystalline and amorphous regions for pure PCL were found to be 35% and 65%, respectively. For PCL-based composite materials containing from 5% to 15% of NCC, these values varied in the range of 40–57% and 60–43%, respectively.

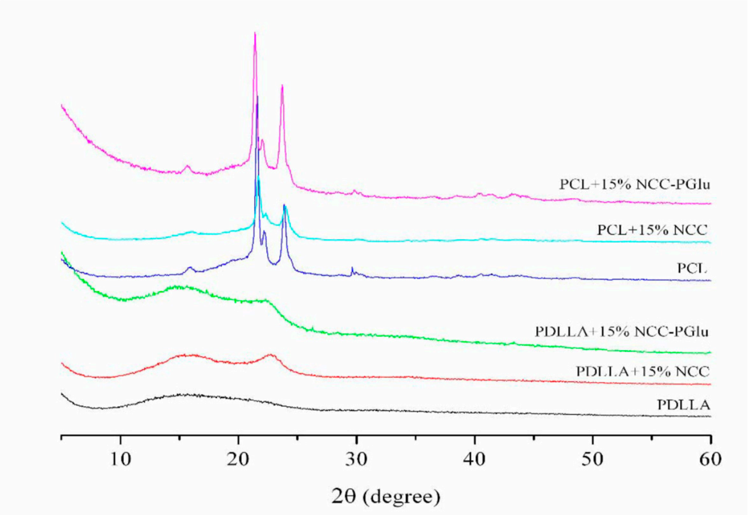

Figure S1. XRD patterns of pure PDLLA and PCL, and their composites with original and modified NCC.

References

1. Korzhikov, V.; Averianov, I.; Litvinchuk, E.; Tennikova, T.B.. Polyester-based microparticles of different hydrophobicity: the patterns of lipophilic drug entrapment and release. *J. Microencapsul*. **2016**, 33, 199–208.
2. Guang-Mei,C.; Tie-Mei, Z.; Lei, C.; Yi-Ping, H. Crystallization properties of polycaprolactone induced by different hydroxyapatite nano-particles. *Asian J. Chem*. **2010**, 22, 5902–5912.

© 2019 by the authors. Submitted for possible open access publication under the terms and conditions of the Creative Commons Attribution (CC BY) license (http://creativecommons.org/licenses/by/4.0/).
